# Insluin and epithelial growth factor (EGF) promote programmed death ligand 1(PD-L1) production and transport in colon cancer stem cells

**DOI:** 10.1186/s12885-019-5364-3

**Published:** 2019-02-15

**Authors:** Mingshui Chen, Aditi Sharma, Yanling Lin, Yanheng Wu, Qi He, Yushu Gu, Zhi Ping Xu, Michael Monteiro, Wenyi Gu

**Affiliations:** 1 Australian Institute for Bioengineering and Nanotechnology (Building 75), The University of Queensland, Cooper Rd., St Lucia, Brisbane, QLD 4072 Australia; 2 Laboratory of Immuno-Oncology, Department of Medical Oncology, Fujian Provincial Cancer Hospital &Institute, Fuzhou, 350014 China; 3 Fujian Provincial Key Laboratory of Translational Cancer Medicine, Fuzhou, 350014 China

**Keywords:** Insulin, EGF, PD-L1, Cancer stem cells, Immunotherapy, PI3K-Akt pathway, Colon cancer

## Abstract

**Background:**

Programmed cell death ligand 1 (PD-L1) is an important immune-inhibitory protein expressed on cancer cells to mediate cancer escape through interaction with PD-1 expressed on activated T lymphocytes (T cells). Previously, we reported that colon and breast cancer stem cells (CSCs) expressed much higher levels of PD-L1 than their parental cells, suggesting they will be more resistant to immune attack.

**Methods:**

We investigated the underlining mechanism of PD-L1 increase in colon CSCs, with a special focus on the effect of insulin and epithelial growth factor (EGF), the two fundamental components to sustain the metabolism and stemness in the culture of CSCs.

**Results:**

We found that insulin increased the total and surface PD-L1 levels through PI3K/Akt/mTOR pathway as the increase could be inhibited by the dual inhibitor of the pathway, BEZ235. EGF didn’t affect the total PD-L1 levels of CSCs but increased the cell surface protein levels by flow cytometry analysis, indicating EGF promotes the transport of PD-L1 to the cell surface. Blocking cell surface PD-L1 with a specific antibody resulted in a significant reduction of tumour sphere formation but didn’t interfere with the sphere growth, suggesting that cell surface PD-L1 may act as an adhering molecule for CSCs.

**Conclusions:**

Apart from the essential roles in metabolism and stemness, insulin and EGF involve in up-regulation of PD-L1 expression in colon CSCs, therefore the inhibition of insulin and EGF/EGFR pathways can be considered for cancer immunotherapy or combined with PD-1/PD-L1 antibody-based cancer immunotherapy to eliminate CSCs.

## Background

Cancer immunotherapy based on blocking immune check-point molecules such as programmed cell death protein 1 (PD-1 or CD279) and its ligand 1 (PD-L1 or CD274 or B7-H1) is a popular topic under intensive investigation. PD-1 expressed on T cells can bind to PD-L1 expressed on cancer cells and leads to the exhaustion of T cells and subsequent survival of cancer cells, also known as immune escape [1–3]. Specific blockade of PD-1 or PD-L1 or both can thus restore T cell function and lead to cancer cell death. Currently, 5 specific antibody agents have been proved by FDA for clinical application [1, 4] and the therapy has achieved exciting outcomes in some advanced cancers including melanoma, renal-cell and non-small-cell lung cancers [5, 6]. However, owing to the dynamic expression and regulation of PD-L1, intratumoral heterogeneity, and the inhibitory tumour microenvironment, the patient response rates to the therapy are limited (20–30%) in many types of solid tumour [2, 7]. This suggests a comprehensive understanding of the mechanism in PD-L1 expression and its regulation in different tumour cell populations is necessary for improving current and developing future PD-1/PD-L1 based immunotherapy for these solid tumours.

Cancer stem cells (CSCs) are a small population of tumour cells with self-renewal and pluri-potent capacities, and are responsible for tumour initiation, maintenance, drug resistance, metastasis and recurrence [8, 9]. Because drug resistance and metastasis/recurrence are the major cause of cancer-related death, targeting CSCs represents a promising strategy to achieve better and long-lasting outcomes including possible prevention of recurrence and metastasis. However, currently, very limited information is available about PD-L1 expression and regulation in CSCs and CSC targeted PD-1/PD-L1 therapy. We previously studied the PD-L1 expression in CSCs from breast cancer MCF-7 and colon cancer HCT-116 cells and showed that PD-L1 expression markedly increased in CSCs for these cancers [10]. But, what factors are responsible for the increase and how they are regulated, especially regarding to the regulation role of fundamental factors in tumour microenvironment such as insulin and epithelial growth factor (EGF) on the expression and transport of PD-L1, were not studied in the study or reported by other researchers.

Insulin and EGF are 2 well-defined metabolic factors having very important biological roles in cell proliferation and differentiation [11, 12]. They also play an essential role in promoting epithelial mesenchymal transition (EMT) and maintaining stemness of cancer cells. For example, insulin, an important regulator for carbohydrate and lipid metabolism and a growth factor for cell proliferation, can keep cancer stemness and EMT of CSCs in hepatocellular carcinoma and breast cancer [13]. EGF, as another important factor involving in cellular proliferation, differentiation, and survival via binding to its receptor EGFR on cell surface, can also promote EMT and maintain stemness in head and neck cancer [12, 14]. Consequently, they both are supplemented as fundamental components in the culture medium to enrich stem cells with self-renewing ability [15] and widely used in the tumour sphere culture to enrich CSCs [16, 17]. However, beside above well-studied functions in metabolism and stemness, their functions in regulating or modulating immune molecules, such as on PD-L1 expression of CSCs, have not been reported before. Understanding their roles in this aspect is essential for the development of more effective and lasting PD-1/PD-L1 cancer immunotherapy targeting CSCs.

It is well-known that pro-inflammatory cytokine IFN-γ regulates PD-L1 expression through JAK/STAT and PI3K/Akt signalling pathways [18–20]. In several cancer types including colon cancer, PI3K/Akt pathway has been highly up-regulated [21–23], this could be a reason for the elevated PD-L1 expression in those cancers. Besides PD-L1 protein production, Ghebeh et al. (2010) reported a doxorubicin-dependent cell surface down-regulation of PD-L1 accompanied by an up-regulation of the protein in the nucleus. This re-distribution of PD-L1 was concurrent with a similar translocation of phosphorylated AKT to the nucleus and inhibition of the PI3K/AKT pathway abrogated the doxorubicin-mediated nuclear up-regulation of PD-L1 [24]. Together, these data indicate that PI3K/AKT pathway is not only involved in upregulating PD-L1 expression but also its translocation to the nucleus. It is known that insulin via its receptor (IR) can perform metabolic activities through PI3K/Akt pathways in stem cells or cancer stem cells [22, 25], we therefore hypothesised that insulin could directly involve in regulating PD-L1 expression or production through PI3K/Akt pathways during the enrichment of CSCs in tumour sphere culture. We would like to test our hypothesis in this study.

For EGF, a study showed that PD-L1 expression increased in an EGFR-dependent manner when EGFR signalling was activated and decreased when EGFR signalling was blocked in esophageal squamous cell carcinoma, and this was through EGFR-PI3K-AKT, EGFR-Ras-Raf-Erk, and EGR-PLC-gamma signalling pathways [26]. Similarly, PD-L1 expression was shown to positively correlate with EGFR expression in non-small-cell lung carcinoma cell lines that were treated in vitro with EGF [27]. They showed that EGFR-tyrosine kinase inhibitors or EGFR small interfering RNA could block EGF-induced PD-L1 expression in the cell lines and that the expression was partially regulated via the PI3K/AKT and JAK/STAT pathways [27]. A recent study further reported that EGF could stabilize PD-L1 via GSK3beta inactivation in basal-like breast cancer and inhibiting EGF signalling by gefitinib destabilized PD-L1 and enhanced anti-tumour efficacy of PD-1 blockade in mouse models [28]. Above data suggest the involvement of EGF/EGFR pathways in PD-L1 up-regulation or stabilization. But whether this is the case in CSCs has not yet reported in any cancers.

In this study, we studied the regulatory roles of these two factors insulin and EGF on PD-L1 expression or transport in CSCs. We found that insulin could increase PD-L1 protein expressions of total and cell surface levels, and this increase was through PI3K/Akt/mTOR pathway. Differently, EGF didn’t directly increase PD-L1 production but increased PD-L1 protein levels on the cell surface, suggesting EGF was involved only in PD-L1 transport or stabilization to the cell surface. These results have important implications for understanding the fundamental metabolism factor in PD-L1 expression in CSCs and the future development of new approaches for PD-1/PD-L1 based immunotherapy targeting CSCs.

## Methods

### Tumour sphere culture

The colon cancer cell line HT-29 was used as a model for the study and was purchased from ATCC (HTB-38) in 2015. The cells were maintained in Dulbecco’s Modified Eagles Medium (DMEM, Invitrogen, Australia) supplemented with 10% foetal calf serum, 1% Penicillin, and 1% streptomycin in 75 ml flasks at 37 °C and 5% CO_2_ and were regularly tested for mycoplasma contamination. Tumour sphere culture method was as previously reported [17]. Briefly, well grown HT-29 cells were seeded in tumour sphere cultural media (Dulbecco’s Modified Eagle’s Medium F-12, 0.4% BSA, 0.2% epidermal growth factor, and 0.2% insulin) at a cell density of 4000 cells/mL in upright T25 flasks. The sphere culture was maintained in a humidified incubator at 37 °C under 5% CO_2_ and fed every two days with 20% of the original culture volume of sphere medium until day 7–8.

### Flow cytometry analysis for colon cancer stemness biomarkers

The spherical cells obtained from sphere culture of HT-29 cells were firstly verified by their surface markers before subsequent experiments. This is to ensure the cancer stem cells have been enriched after sphere culture. The tumour spheres were treated with 1:1 diluted trypsin (2.5% Trypsin-EDTA, Invitrogen, Australia) for 5 min at 37 **°**C and washed. Spherical cells were dissociated by repeated pipetting and passing through a cell strainer (40 μM, Falcon, USA). After dissociation, 1 × 10^5^ cells were used for staining with rabbit anti-human CD133 (prominin-1) antibody (Sigma-Aldrich); mouse anti-human CD44 conjugated with FITC (Invitrogen, Australia); and mouse anti-human CD24 antibody conjugated with RPE (Invitrogen, Australia). For CD133 staining, mouse anti-rabbit IgG-FITC (Sigma-Aldrich) was used as the secondary antibody. After 3 washes with 2% FCS/PBS, the cells were fixed in 2% paraformaldehyde/PBS and analysed by flow-cytometry (Accuri, BD) and CFlow Sampler software.

### Insulin and EGF treatment to HT-29 cells

HT-29 cells were cultured in DMEM media with 10% foetal calf serum (FCS) for 4–6 h in 6-well plates to allow cell attachment. After the attachment, cells were washed with DMEM media without FCS and then added DMEM media with 4% FCS in the presence of 1μg/ml, 5μg/ml, 10μg/ml, 20μg/ml and 40μg/ml insulin and EGF, respectively. The cells were incubated for 3 days or 6 days and collected for PD-L1 expression by flow cytometry or in RIPA buffer (Cell Signal Technology) for Western blotting assays of PD-L1 protein expression.

### Western blotting analysis

Cells from cultures were lysed in RIPA buffer (Cell Signal Technology) containing 2 μl/ml protease inhibitor cocktail (Sigma-Aldrich). Protein samples were separated by electrophoresis using pre-casted mini PAGE (Bio-Rad) at 120 V for 1.5 h. The separated proteins were transferred onto PVDF membrane at 100 V for 1 h. The membrane was blocked at room temperature with 5% bovine serum albumin in Tris-*Buffered* Saline and 0.5% Tween 20 (TBST) buffer for 1 h and washed three times with TBST with each wash being 5 min. The membrane then was incubated overnight with rabbit anti-human PD-L1 antibody (Cell Signal Technology) at 1:500 dilution. After washing three times with TBST, the membrane was incubated for 2 h at room temperature with horseradish peroxidise conjugated goat anti-rabbit antibody (Cell Signal Technology) at dilution 1:2500. The membrane was incubated with ECL for 5 min and imaged by GelDoc UV illuminator (Biorad Laboratories).

### PI3K-Akt /mTOR pathway dual inhibitor BEZ235 treatment

To investigate the effect of insulin on PD-L1 expression in HT-29 cells through PI3K/Akt pathway, HT-29 cells were cultured in complete DMEM medium for overnight. After attachment cells were washed with DMEM and treated with 50 nM and 100 nM of PI3K/Akt inhibitor BEZ-235 respectively for 4 h, then cells were maintained at 37 °C 5% CO2 in the presence of 4μg/ml insulin for 3 or 6 days. On day 3 or 6 cells were collected and lysed in RIPA buffer for PD-L1 protein expression or for flow cytometry analysis. HT-29 cells cultured in DMEM and DMEM in the presence of 4 μg/ml insulin, respectively, served as controls.

### PD-L1 antibody blocking assay in sphere culture

To investigate PD-L1 antibody block effect on sphere formation and growth, HT-29 cells were cultured in sphere culture medium supplemented with anti-PD-L1 antibody (Cell Signalling Technology) at a concentration of 0.08 μg/ml on day 1. On day 4, an additional 1 ml of sphere culture medium with anti-PD-L1 antibody was added to the culture. The culture continued for another 3 days. On day 7 of culture, the spheres were harvested by gentle centrifugation and the sphere number was counted under a microscope. The effect of PD-L1 antibody on cell growth was assessed by sphere size. To determine the size of spheres, spheres were collected by gentle centrifugation and trypsinized to separate individual spherical cells. Cell number were counted using hemocytometer under a microscope. Sphere size was defined as cell number per sphere in average (total spherical cells/ sphere number).

### PD-L1 protein analysis on cell membrane

To study if EGF plays a role in transferring PD-L1 protein to cell membrane, HT-29 cells were cultured in DMEM medium supplemented with 5μg/ml insulin. On day 6, EGF at 20 μg/ml was added in the culture for 24 h. On day 7, cells were collected to extract membrane protein for Western blotting of PD-L1 expression. Cells treated with 5μg/ml insulin and 20 μg/ml EGF alone for 7 days served as controls. Extraction of membrane protein was as previously described with minor modifications [29]. Briefly, cells were harvested by centrifugation and re-suspended in homogenization buffer and were sonicated for 20 s on ice. A volume of 6.6 ml homogenizer was transferred into 10 ml ultracentrifuge tubes and under-layered with 2.6 ml 40% sucrose solution. The tubes were centrifuged 96,000 X g for 1 h at 4 °C. The interfaces were recovered and transferred into 50 ml tube and then was diluted to 20 ml with PBS. After another centrifugation, the supernatant was discarded, and the precipitation was re-suspended with 100 ul PBS and was used for Western blotting to detect PD-L1 protein.

### Data analysis

Data collected from experimental and control groups with at least 3 biological repeats were expressed as mean ± SD (*n* = 3). Unpaired Student’s *t*-test (GraphPad Prism 7 program) was used to analyse the differences between experimental and control groups (two-tails, *P* < 0.05 was considered significance).

## Results

### Characterizations of spherical cells from colon cancer HT-29 cells

**Fig. 1:**
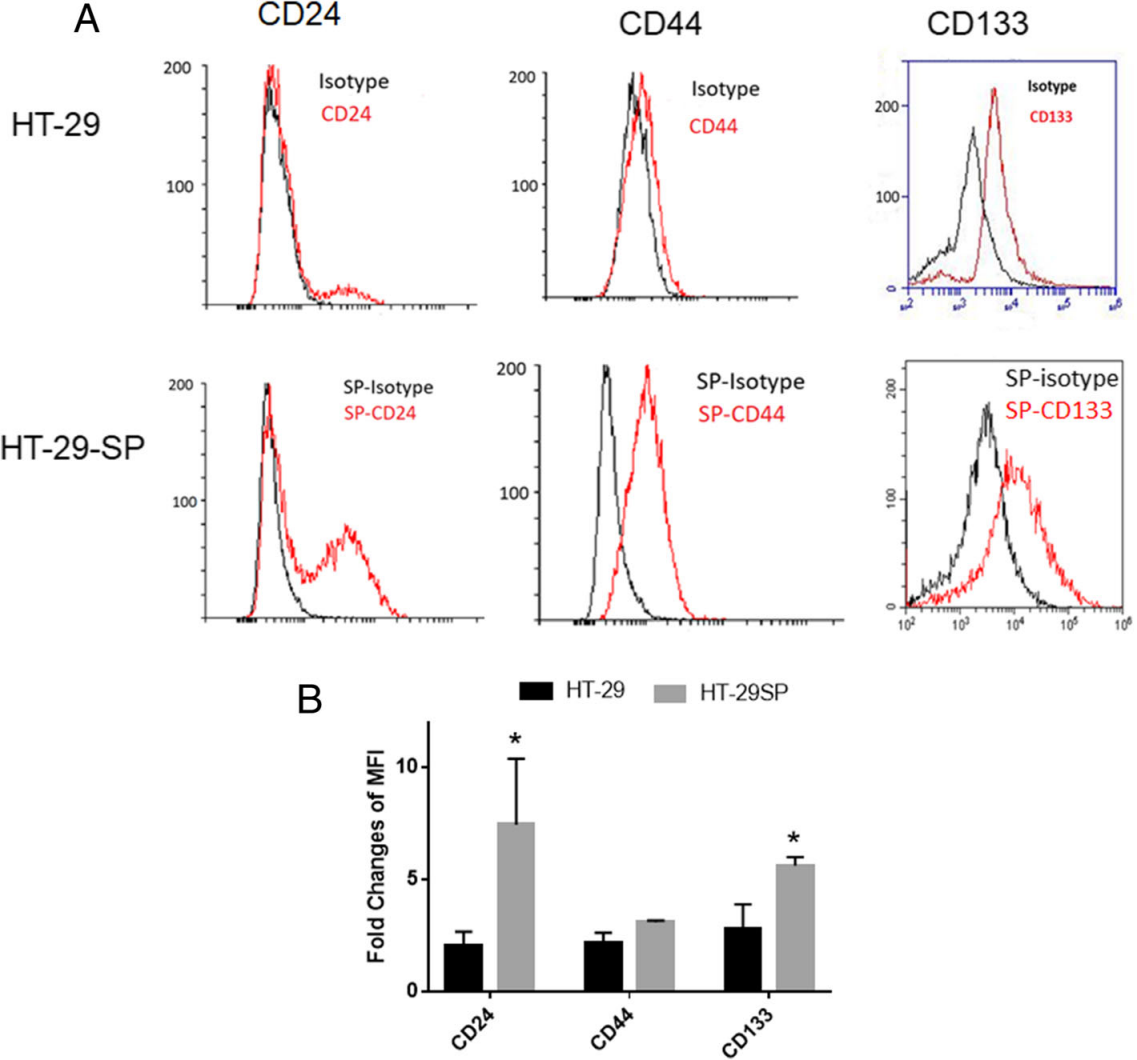
Flow cytometry analysis of CSC markers of HT-29 spherical cells. After sphere culture for enrichment of cancer stem cells, the HT-29 spherical (SP) cells were labelled and analysed for the expression of CD24, CD44, and CD133 using flow cytometer. The expression of CD24, CD44, and CD133 of HT-29 spherical cells were significantly increased (**a**, HT-29 vs HT-29SP). The fold changes of mean fluorescent intensity (MFI) from 3 repeated FACS analysis show the same trend (**b**)

Tumour sphere culture was widely used to enrich CSCs from cancer cell lines or primary cancer cells [16, 17]. Using this method, we previously enriched CSCs from colon cancer HCT-116 cells [10, 22]. In this study, we used the same method to enrich colon CSCs from colon cancer cell line HT-29. After 6–7 days of culture, the tumour spheres formed, and they were harvested. The spherical cells were disassociated by treatment with trypsin and passed through 40 μm cell strainers. The cells were assayed for characteristic surface markers for colon CSCs and compared with parental HT-29 cancer cells. The results showed that after sphere culture, the spherical cells express higher levels of CD24, CD44, and CD133 than HT-29 cancer cells (Fig. 1a, b, and c). The high levels of these markers reflect the characteristics of colon cancer stem cells reported by us and others [10, 22, 30].

### PD-L1 expression increased in colon CSCs from HT-29 cells

**Fig. 2:**
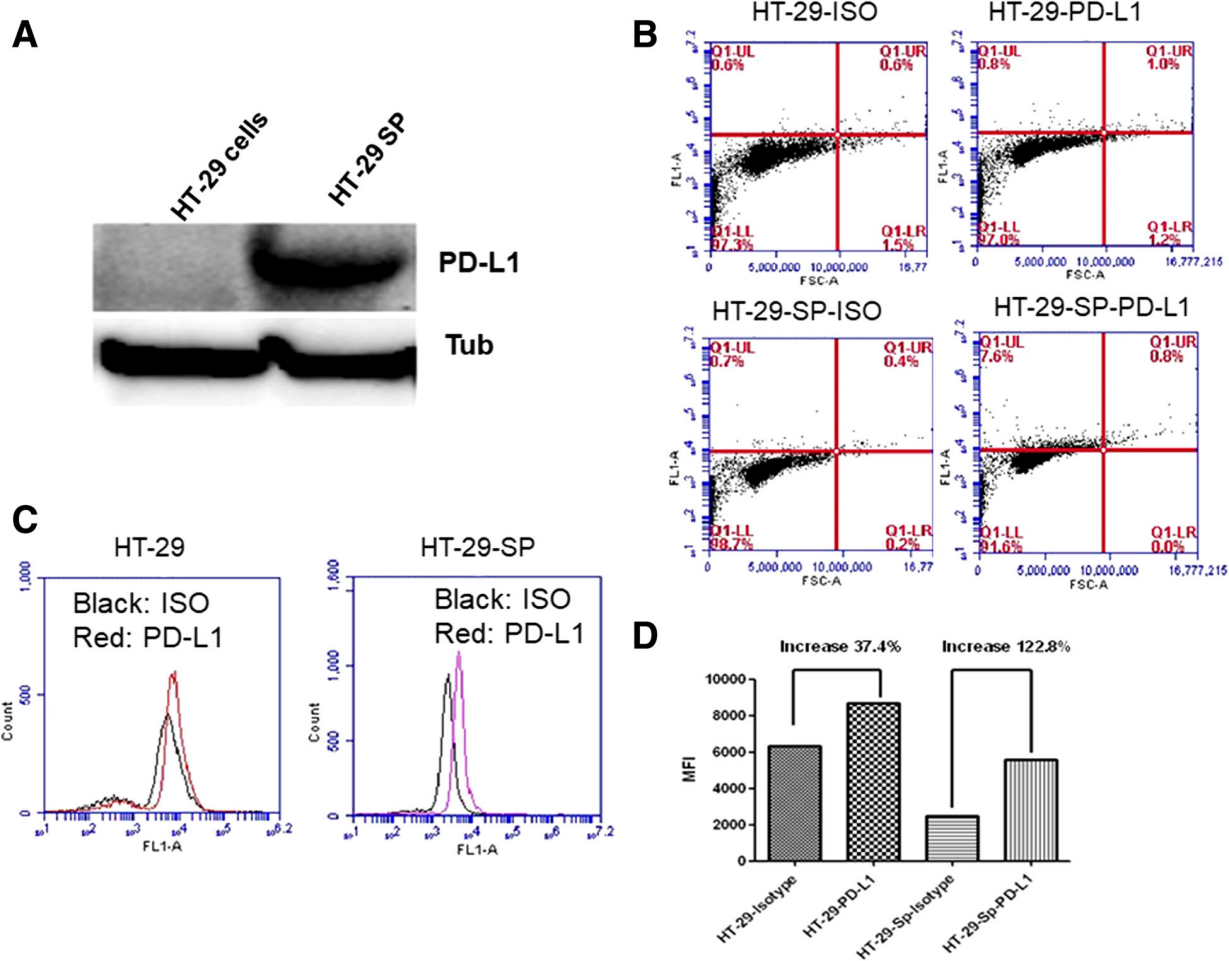
PD-L1 expression increased in CSCs from HT-29. **a**, Western Blotting analysis of PD-L1 protein levels in HT-29 cancer cells and tumour spherical cells from HT-29 (HT-29 SP). **b**, flow cytometry analysis of PD-L1 positive cells. Comparing with the isotype controls (ISO) HT-29 spherical cells (HT-29 SP) had more (about 10% increase of PD-L1 positive cells. **c**, a histogram analysis of PD-L1 expression in HT-29 cells and HT-29SP. **d**. the fold changes of MFI from **c**

The spherical cells with enriched CSCs were then assayed for PD-L1 expression. The Western blotting assay results showed that PD-L1 total protein levels were dramatically increased in CSCs, compared to HT-29 cells (Fig. 2a). The flow cytometry analysis further proved that the surface PD-L1 expression also increased. The data showed that in enriched CSCs, PD-L1 positive cell percentage increased over 8% compared to HT-29 cells (Fig. 2b) but the mean fluorescent intensity (MFI) of these spherical cells increased much more significantly (Fig. 2c, d), indicating that after enrichment of CSCs, there was a global increase of PD-L1 expression in these cells. These data in HT-29 cells with our previous results in colon cancer HCT-116 cells [10] together suggest that PD-L1 expression increase is more common in colon CSCs and this increase includes both surface protein and total protein production in these cells.

### Insulin but not EGF increases total PD-L1 protein production

**Fig. 3:**
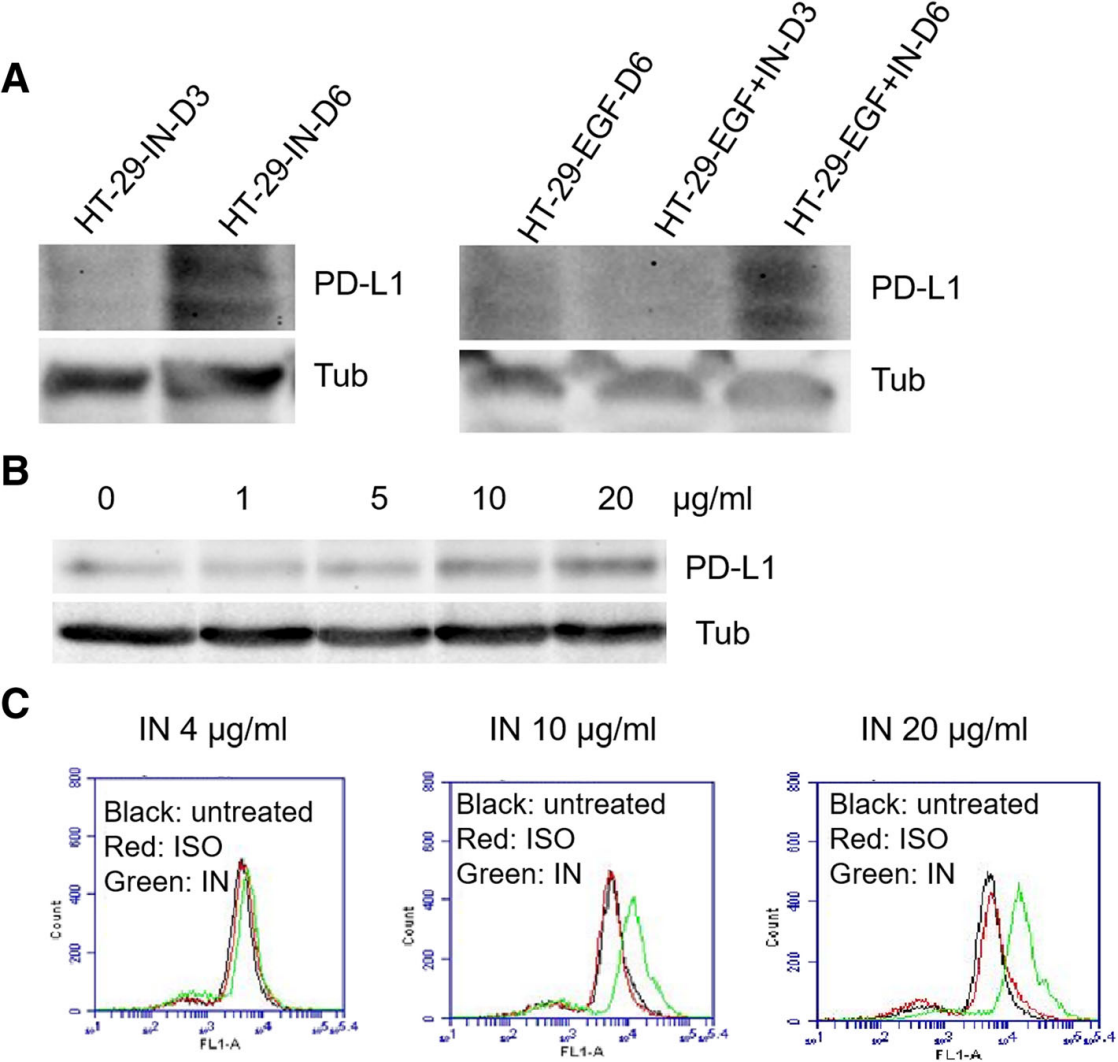
Insulin but not EGF increases PD-L1 expression in non-CSCs. Insulin treatment can increase PD-L1 expression in non-cancer stem cells of HT-29 but the treatment needs to be 6 days to be able to display by Western blotting assay (**a**). In contrast, EGF treated HT-29 didn’t show PD-L1 protein band even after 6 days and only have the band after co-treatment with insulin. **b** shows a dose-dependent increase of PD-L1 protein from 1μg/ml to 20 μg/ml insulin. Like **b**, **c** shows the dose-dependent increase of PD-L1 by flow cytometry analysis

To identify if insulin or EGF or both are responsible for the PD-L1 increase in CSCs, we treated HT-29 cells individually or together for 6 days. We measured the PD-L1 protein levels at day 3 and day 6. Our Western blotting (WB) results showed that only insulin treatment for 6 days at the same dose as in sphere culture significantly increased PD-L1 protein levels in HT-29 cells (Fig. 3a). In contrast, EGF treatment at the same dose did not increase the PD-L1 level at either day 3 or 6 post treatment (Fig. 3a). To further confirm the effect of insulin we carried out a dose-dependent assay, our data showed that insulin could increase PD-L1 protein levels in a dose-dependent manner in HT-29 cells (Fig. 3b). To further confirm cell surface expression, we used flow cytometry analysis and found that insulin also increased PD-L1 expression on cell surface in a dose-dependent manner (Fig. 3c). These data collectively suggest that insulin is responsible for promoting PD-L1 production and surface expression in CSCs.

### EGF helps PD-L1 transport to cell membrane

**Fig. 4:**
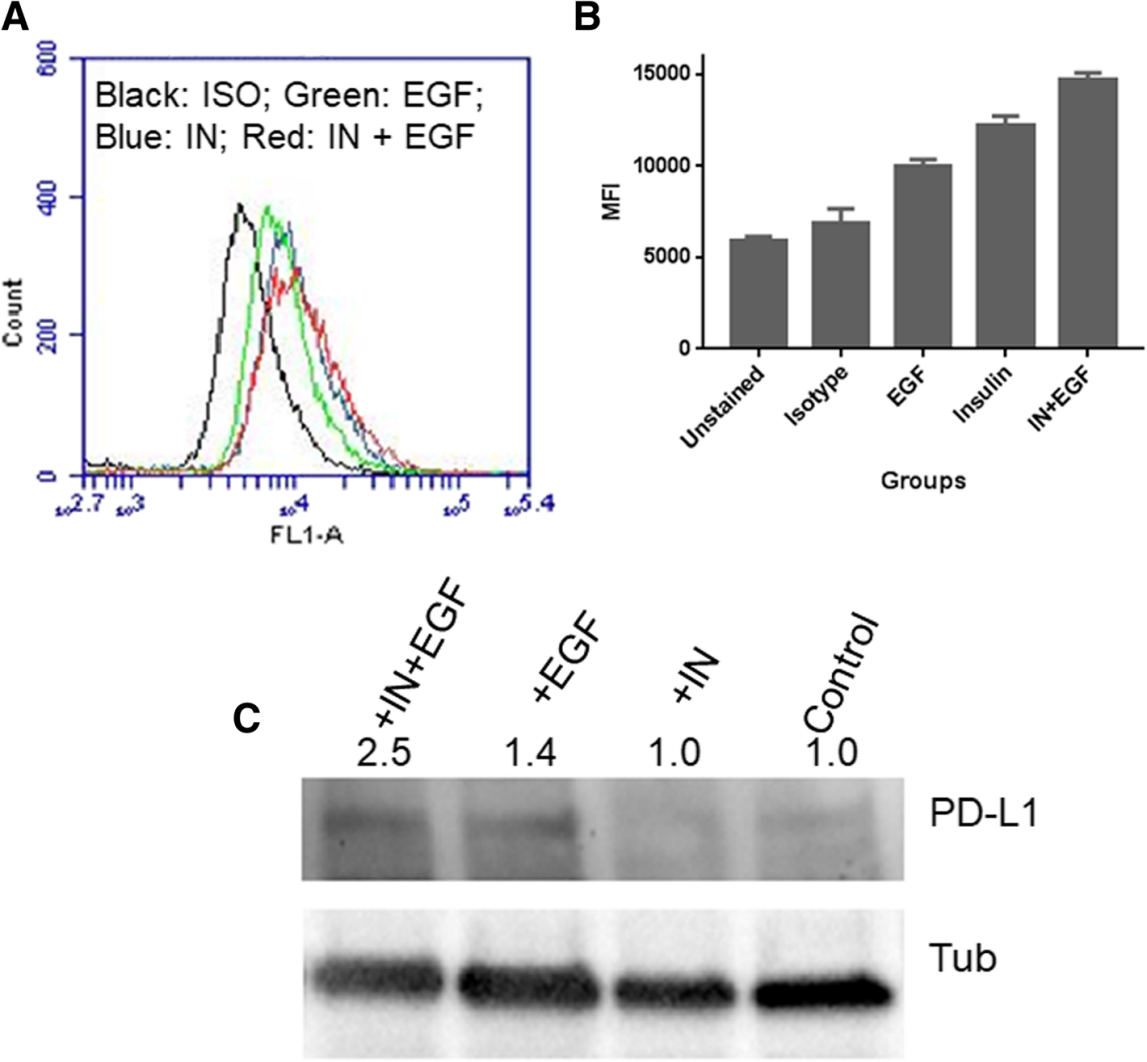
EGF helps in PD-L1 transport to cell surface. **a**, shows that after EGF treatment the PD-L1 levels on cell surface increases analysed by flow cytometry analysis, compared to isotype control. Together with insulin EGF treatment can further increase the level. **b**, shows the MFI of repeated flow cytometry analysis. EGF treatment alone the MFI increased 44.7%, compared to isotype control. When co-treated with insulin, the MFI of EGF treatment further increased 20.4%, suggesting that EGF treatment is associated with membrane PD-L1 levels. **c**, shows the WB assay for PD-L1 in the cell membrane isolated from HT-29 cells treated with insulin or EGF+ insulin

Though we did not observe the increase of total protein after EGF treatment by WB assays, we found an increase of PD-L1 in the EGF treated cells when we analysed the cells by flow cytometry (Fig. 4a). This suggests that EGF may play a role in transporting PD-L1 to the cell surface. We have repeated flow cytometry assay many times and obtained the similar results (Fig. 4b). A previous study showed that EGF could enhance PD-L1 expression by stabilising membrane PD-L1 [27]. Therefore, we further investigated if EGF treatment could increase PD-L1 levels in cell membrane. We isolated the cell membrane of EGF treated cells and conducted WB. We found that EGF treatment increased PD-L1 proteins in cell membrane, and together with insulin treatment it increased even more (Fig. 4c). These data indicate that EGF plays a role in PD-L1 transport to cell membrane, assuming mainly transfer PD-L1 from cytoplasm to membrane and stabilise the protein on the membrane, as the previous study suggested [28].

### Insulin increases PD-L1 expression through PI3K/Akt pathway

**Fig. 5:**
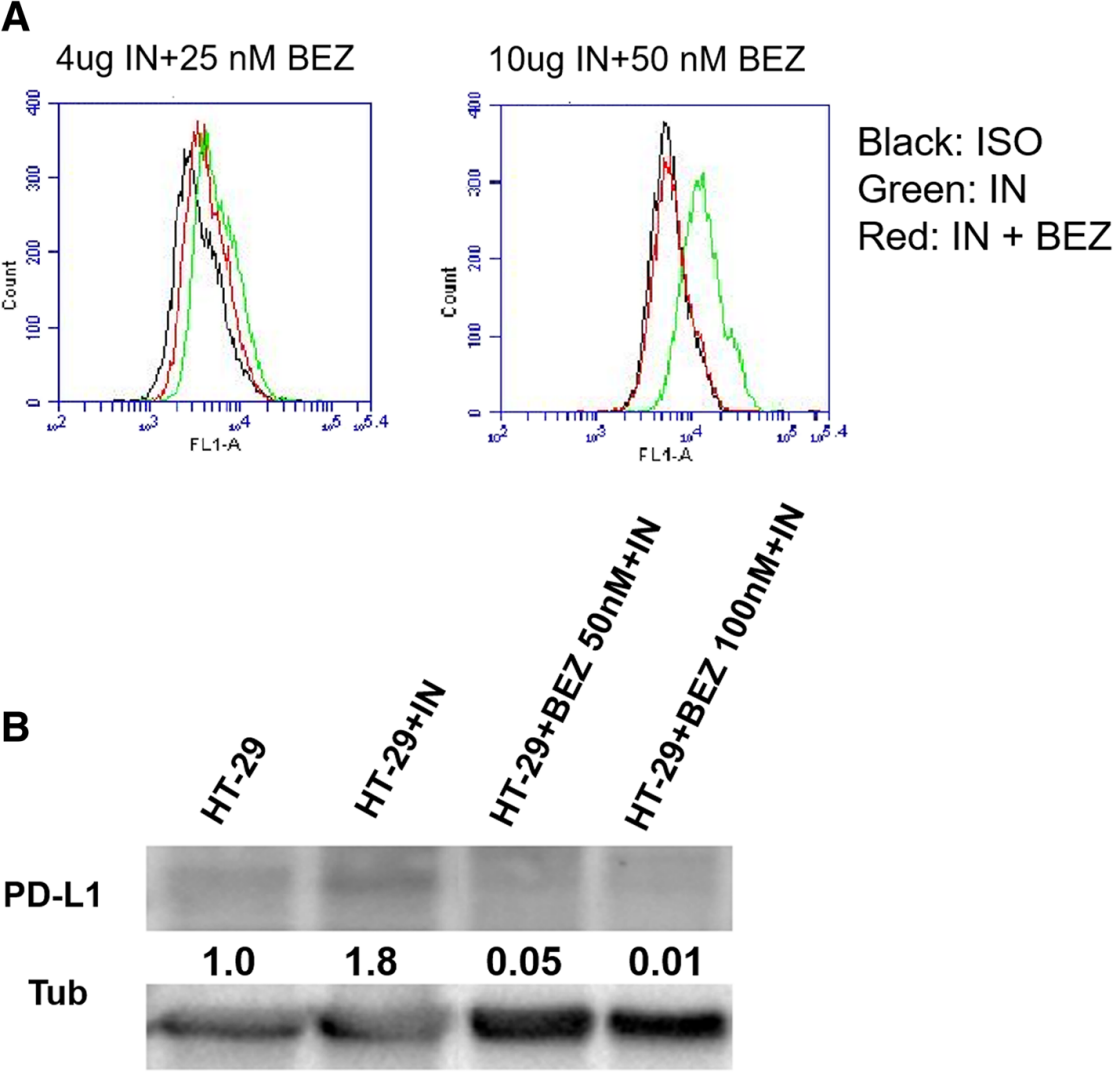
PI3K/Akt dual inhibitor BEZ235 dose-dependent inhibition of insulin-induced PD-L1 expression. **a**, shows flow cytometry results of BEZ235 inhibitory effect on PD-L1 expression. **b** shows the inhibitory effect on protein levels of BEZ235 by Western blotting assay

To understand whether insulin promotes PD-L1 expression is through PI3K/Akt/mTOR pathway, we employed a dual inhibitor of this pathway BEZ235 to examine if it can inhibit the promotional effect of insulin on PD-L1 expression. Indeed, our data showed that BEZ235 treatment could inhibit PD-L1 expression at 25 and 50 nM concentrations by flow cytometry assays (Fig. 5a). Our WB assays further confirmed the results in total protein levels (Fig. 5b). These data confirm our hypothesis that insulin promote PD-L1 protein production and expression through PI3K/Akt/mTOR pathway.

### Blocking PD-L1 on CSC surface only affects sphere formation

**Fig. 6:**
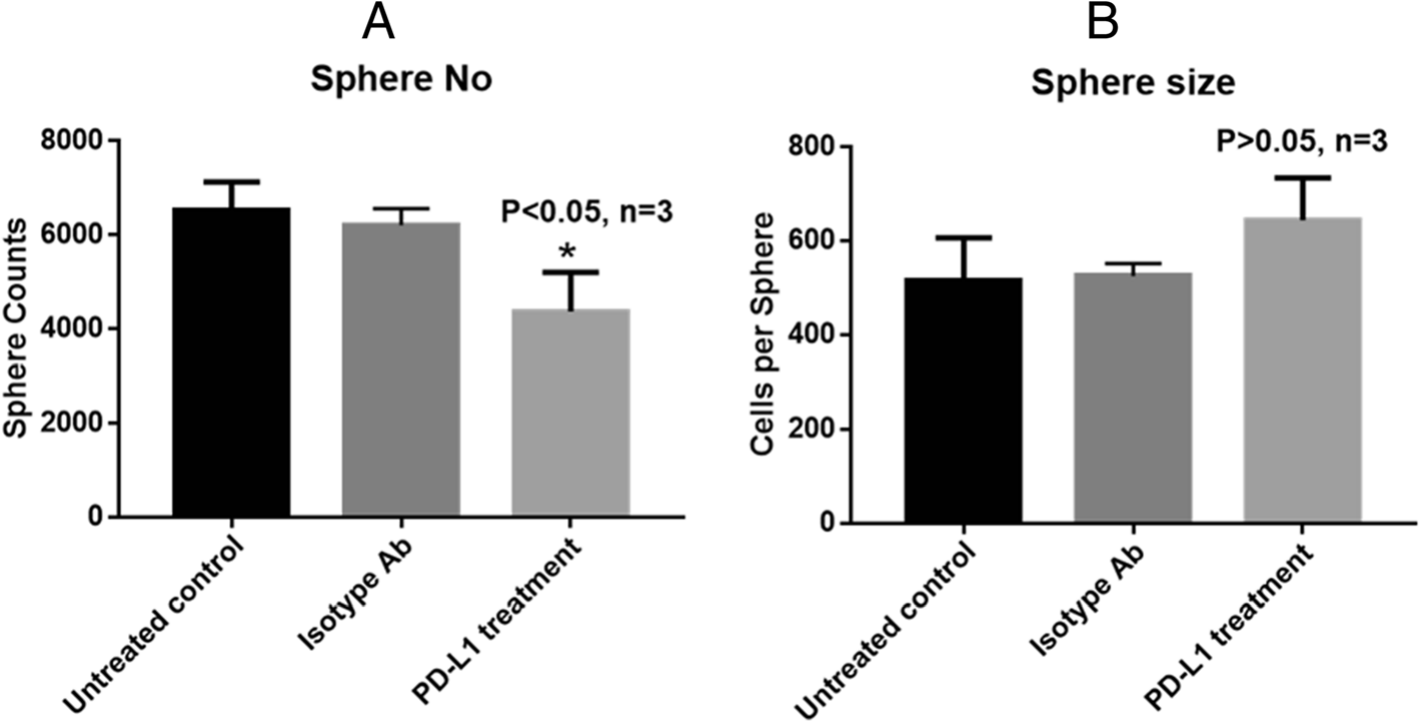
Blocking surface PD-L1 inhibits tumour sphere formation. Antibody blocking PD-L1 or isotype antibody (Isotype Ab) was added into the sphere culture medium and the blockage of PD-L1 inhibited the sphere number (*P* < 0.05, **a**), compared to both untreated and isotype controls. However, the antibody blocking didn’t affect the sphere size (cells/sphere, **b**)

To explore whether the high-level surface expression of PD-L1 have any biological function in colon CSCs. We used the PD-L1 specific antibody to block PD-L1 in the sphere culture system and examine if the block interferes with sphere formation and spherical cell growth. The results showed that addition of the antibody significantly reduced the tumour sphere numbers (Fig. 6a) but did not affect the sphere size (Fig. 6b). The sphere formation reflects the cancer cell clustering ability while the size (determined by average cell number per sphere) reflects the cell growing and self-renewal ability. Our data suggest that the blocking of PD-L1 on CSCs only interfered with their clustering ability but not cell growing or self-renewal ability.

## Discussion

Insulin and EGF are 2 well-understood growth factors for cell proliferation and differentiation. Their functional roles in promoting cancer stemness are also well-known. However, whether they could regulate the expression of immune inhibitory molecules on CSCs was not clear. In this study, we studied both factors on PD-L1 expression in colon CSCs and demonstrated that during their promotion of the stemness in colon CSCs they increased PD-L1 production and transport in these cells. As a result, the CSCs expressed substantial high-levels of total and cell surface PD-L1 than the parental cancer cells and PD-L1 became a part of cancer stemness. A previous study reported that surface PD-L1, E-cadherin, CD24, and VEGFR2 were markers of epithelial cancer stem cells associated with rapid tumorigenesis [31], supporting our assumption. To extend to in vivo setting, because the two factors can be well present in/around tumour [14, 32], they should be considered as essential factors to promote PD-L1 expression in CSCs in at least colon cancer. This also suggests that high-levels of both insulin and EGF in tumour microenvironment can be a risk factor for cancer escape.

In this study, we showed that CSCs from colon HT-29 cells expressed high levels of PD-L1, this together with our previous study on colon cancer HCT-116 cells and breast cancer MCF-7 cells [10] reveal a more common feature of epithelial CSCs expressing high-levels of this protein. Consequently, insulin and EGF induced PD-L1 high expression may be also common in epithelial cancers. Previous studies reported that the increased levels of PD-L1 in cancer patients with different types of tumours were associated with poor prognosis [33–35]. We assume that the high-levels of PD-L1 in CSCs will attribute to this, as CSCs are responsible for drug resistance, tumour recurrence and metastasis, and now immune escape. From this aspect, CSCs should be targeted in cancer immunotherapy for lasting and better treatment outcomes. On the other hand, the reason for PD-1/PD-L1 based immunotherapy has achieved promising results in some advanced cancers might be owing to the therapy has targeted CSCs in these cancers, because advanced tumour would generally contain more CSCs. Therefore, understanding the molecular mechanism of PD-L1 regulation in CSCs has important scientific and practical implications for future development of more effective and lasting PD-1/PD-L1 immunotherapy. Previous reports showed that only a few melanoma and thymic cancer cases had 100% positive cells for PD-L1 expression, most cancer types had only < 60% positive PD-L1 cells [36]. For these cancers, the detection of PD-L1 expression in CSCs may be more useful for guiding whether a PD-1/PD-L1 based immunotherapy should be conducted.

Besides immunological functions, the high-level PD-L1 expression in CSCs seems necessary for maintaining cancer stemness. In this study, our data suggest that antibody blocking of surface PD-L1 of CSCs can reduce sphere formation rate but not sphere growth (Fig. 6), indicating surface PD-L1 may play a role like the adhering molecule for cell-cell connection rather than involving in signalling pathways for cell survival and growth. A recent study showed that down-regulation of intrinsic PD-L1 compromised the self-renewal capability of breast CSCs in vitro and in vivo, as shown by tumour sphere formation assay and extreme limiting dilution assay, respectively [37]. In the same study it was demonstrated that PD-L1 promoted OCT4 and Nanog expression in breast CSCs, which are stem cell markers [37]. Combining this study with our data, we can conclude that: 1) there is a new function role of PD-L1 in sustaining cancer stemness in different types of cancer. The implication of this is that for PD-1/PD-L1 based therapy it can not only enhance anti-tumour immunity but also inhibit CSC activities; an obvious advantage of the immunotherapy. However, since CSCs are usually located in deep tumour niche, the tumour microenvironment may protect CSCs from attack by anti-PD-L1 antibody and T cells [38] thus for an effective treatment, a delivery system will be necessary for breaking the barrier and delivering its therapeutic load; 2) Apart from cell surface, PD-L1 distributed in other cell compartments needs to be considered in the therapy, particularly these in cytoplasm and nucleus with possible different functions. Indeed, from literature, PD-L1 proteins are found in membrane [39–42], cytoplasm [40, 42, 43], nuclear [24, 44], and soluble format [45] from different cancer types. These studies also showed that the increased cytoplasm, nuclear, and even soluble PD-L1 levels are correlated with poor prognosis. But it is expected that only membrane and soluble PD-L1 will be blocked by PD-L1 antibody therapy. How the PD-L1 proteins in cytoplasm and nucleus relates to the membrane PD-L1 and their activities in cancer cells after blocking the membrane PD-L1 are interesting topics for investigation and a good understanding of the relationship between PD-L1 distributed in cellular locations will lead to better strategies to more effectively block PD-L1 functions in cancer cells. A previous study reported that the increased cytoplasmic PD-L1 in all four stages of papillary thyroid carcinoma patients was correlated with a greater risk of recurrence and a poor prognosis, but increased membrane PD-L1 significantly correlated with a greater risk of metastasis or death in stage IV patients [43], supporting that both intracellular and surface PD-L1 are playing an important role in tumour development and metastasis thus need to be considered together in the therapy.

Insulin is an important regulator for cell metabolism and growth, it also plays a role in keeping cancer stemness and epithelial mesenchymal transition (EMT) of CSCs [13]. In the tumour sphere culture system, insulin is an essential factor to promote the growth and stemness for enriching stem cells [15, 16]. In this study, to be sure that this was not just in the sphere culture system, we also tested insulin treatment in adherent 2-D culture of HT-29 cells. We observed a dose-dependent increase of PD-L1 protein expression (Fig. 3b), suggesting that insulin can generally stimulate PD-L1 production in colon cancer cells. To the best of our knowledge, this is the first evidence on insulin promotion of PD-L1 production.

It is well-known that PIK3/Akt pathway regulates PD-L1 expression [19, 21] and it has also been reported that insulin can activate this pathway in colon CSCs [22]. Therefore, in this study we tested if the dual inhibitor of this pathway BEZ235 could stop insulin induced PD-L1 production. We indeed confirmed that insulin increased PD-L1 production through PI3K/Akt pathway by using both the Western blotting and flow cytometry analysis (Fig. 5). However, our data also showed that insulin promotion required a long period (between 3 to 6 days), which was a similar period for the sphere culture. The reason behind this is not clear, one possible explanation is that PI3K/Akt pathway has been highly activated in colon cancer [22], and further activation of this pathway needs take a longer time. Another possible explanation is that CSCs grow slower than non-CSCs thus the signalling pathway activation and PD-L1 synthesis take a longer time. Or an alternative explanation is that other pathways such as hypoxia related HIFs [45] production may promote the activation of these pathways, which takes time. Further studies of these processes are needed to fully understand the mechanism. Nevertheless, the inhibition of PI3K/Akt/mTOR pathway may be an important strategy for PD-1/PD-L1 based immunotherapy. The dual inhibitor, BEZ235 can inhibit not only the over-expressed PI3K/Akt/mTOR signalling pathway in colon cancer cells but also the PD-L1 expression, the later can then increase the sensitivity of cancer cells to T cell-mediated killing. We previously showed that BEZ235 had a remarkable effect on colon CSCs [22]. Form this point of view, BEZ235 will be a good dual action chemotherapeutic for at least colon cancer treatment. In addition, a combination therapy of anti-PD-L1 antibody with BEZ235 to CSCs may achieve an even better outcome.

Different from insulin, EGF didn’t directly affect PD-L1 production. A previous study showed that EGF could stabilize PD-L1 via GSK3beta inactivation in basal-like breast cancer [28]. However, a recent study reported that EGF was associated with PD-L1 transport, in which, the PD-L1 expression in cancer cell lines of non-small cell lung carcinoma (NSCLC) was shown to be enhanced by EGF treatment using flow cytometry analysis, and inhibition of EGFR by EGFR-tyrosine kinase inhibitors or EGFR small interfering RNA (siRNA) blocked EGF-induced PD-L1 overexpression in NSCLC cell lines [27]. They also showed that PD-L1 expression was partially regulated via the PI3K/AKT and JAK/STAT pathways and concluded that PD-L1 overexpression was positively correlated with EGFR expression in NSCLC [27]. Supporting this study, Zhang W et al. (2017) reported that in oesophageal squamous cell carcinoma EGF/EGFR was involved in increasing surface PD-L1 expression using flow cytometry method [26]. From these studies, it seems that EGF/EGFR play a transporting or stabilizing role in PD-L1 regulation. In our present study, we used both Western blotting and flow cytometry methods to analyse PD-L1 expression and demonstrated that EGF treatment only led to the protein increase on cell surface, suggesting that EGF promote the transport or stabilization of PD-L1 on cell surface. This is further confirmed by our membrane protein WB analysis (Fig. 4c).

**Fig. 7:**
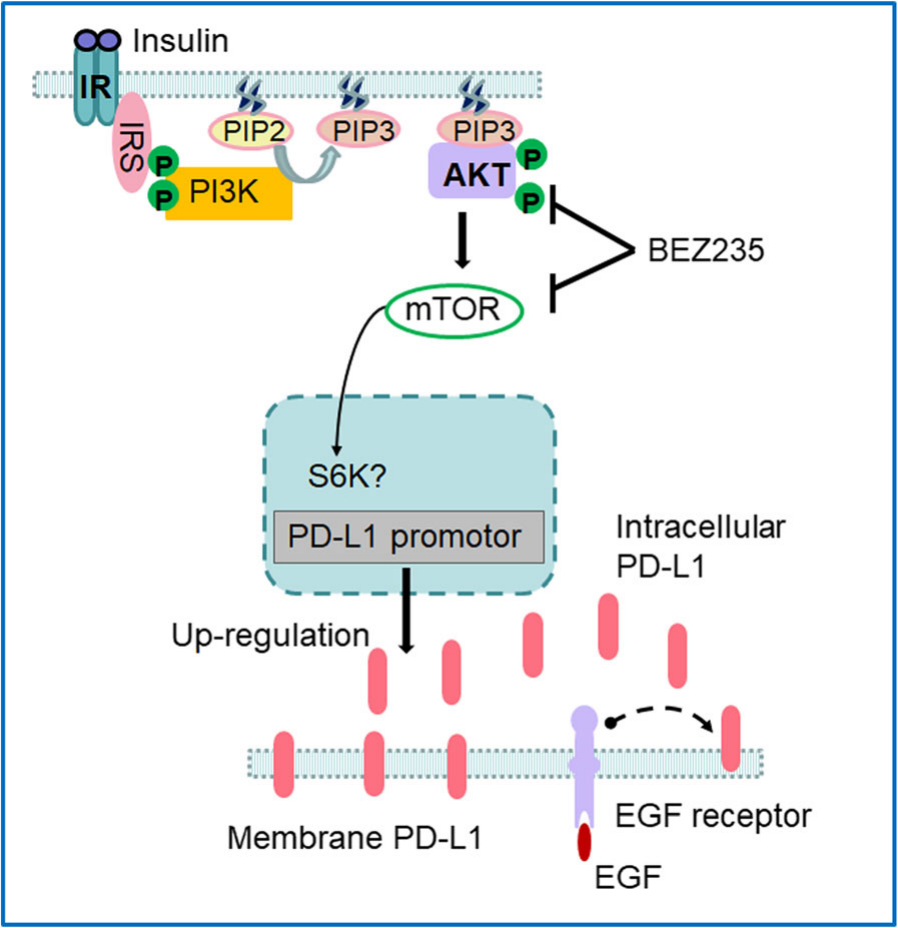
Schematic diagram illustrating insulin-induced PD-L1 up-regulation and EGF help in PD-L1 transport in colon CSCs. Insulin binds to its receptor IR and activates the IRS which phosphorylate the PI3K. Activated PI3K can activate AKT through PIP3 and phosphorylate AKT. A downstream target of AKT is mTOR, that can lead to activation and translocation of possible S6K into the nucleus [46, 47] and subsequent start of the PD-L1 transcription and PD-L1 protein production. Cytoplasmic PD-L1 proteins will be transferred to cell membrane through EGF/EGFR-mediated transport or stabilization on the cell membrane. Because BEZ235 can inhibit both AKT and mTOR thus reduce PD-L1 production via this pathway

Furthermore, our flow cytometry data showed that comparing to insulin treatment alone, the insulin + EGF treatment increased surface PD-L1 levels even more (Fig. 4a, b). This result supports that EGF plays a role to transfer PD-L1 to the cell membrane when insulin has promoted more PD-L1 protein production in cytoplasm, as if EGF only plays a stabilising role, the membrane PD-L1 level may not always correlate with the cytoplasm protein level. This notion is also supported by a previous study by Okito R et al. (2017) where they observed when PI3K/Akt pathway was activated and PD-L1 was produced, the surface PD-L1 increased by flow cytometry analysis, when the pathway was blocked, PD-L1 production reduced and surface PD-L1 transport also decreased [27]. Our result on cellular membrane PD-L1 detection also supports the transport role of EGF as the most increased membrane PD-L1 protein level was seen in the group treated with both insulin and EGF but not EGF alone. Taken together, all data above suggest that insulin promote PD-L1 protein production through PI3K/Akt/mTOR pathways and EGF promote its transport in colon CSCs through proposed pathways (Fig. 7).

## Conclusions

In this study, we showed that colon CSCs from HT-29 cells expressed high-level PD-L1 protein in both cell membrane and cytoplasm. Insulin promotes its expression through PI3K/Akt pathway and EGF helps in its transport to membrane. Together they increase intracellular and cell surface PD-L1 expression in CSCs (Fig. 7). The high-level PD-L1 expression in CSCs is associated with their sphere formation ability and possible stemness. The current data together with our previous study in colon cancer HCT-116 cells and breast cancer MCF-7 indicates that it is common in some cancer types that CSCs express higher level PD-L1 than differentiated cancer cells. This suggests that PD-1/PD-L1 based therapy targeting CSCs can be more effective and lasting in these cancers. In addition, as HCT-116 cells are K-Ras mutated cells while HT-29 cells are wild type cells, our data reveal that K-Ras mutation may not significantly affect PD-L1 expression in colon cancer. For EGF transport role, blocking EGF/EGFR signalling pathway to reduce PD-L1 transport in CSCs may be an alternative strategy to enhance anti-tumour immune response.

## Abbreviations

AKT: Protein Kinase B
EGF: Epithelial growth factor
EGFR: EGF receptor
EMT: Epithelial mesenchymal transition
JAK/STAT pathway: Janus kinase/signal transducers and activators of transcription pathway
mTOR: Mechanistic target of rapamycin
PD-1: Programmed death protein 1
PD-L1: programmed death protein ligand 1
PI3K: Phosphoinositide 3-kinase
VEGFR2: Vascular endothelial growth factor receptor 2
